# Salt-Assisted Ultrasonicated De-Aggregation and Advanced Redox Electrochemistry of Detonation Nanodiamond

**DOI:** 10.3390/ma10111292

**Published:** 2017-11-10

**Authors:** Sanju Gupta, Brendan Evans, Alex Henson, Sara B. Carrizosa

**Affiliations:** 1 Department of Physics and Astronomy and Biotechnology Center, Western Kentucky University, Bowling Green, KY 42101, USA; brendan.evans054@topper.wku.edu (B.E.); alex.henson836@topper.wku.edu (A.H.); 2 Department of Chemistry, Western Kentucky University, Bowling Green, KY 42101, USA; sara.boterocarrizosa090@topper.wku.edu

**Keywords:** detonation nanodiamond, electrochemistry, surface redox chemistry, scanning electrochemical microscopy, energy band structure

## Abstract

Nanodiamond particles form agglomerates in the dry powder state and this poses limitation to the accessibility of their diamond-like core thus dramatically impacting their technological advancement. In this work, we report de-agglomeration of nanodiamond (ND) by using a facile technique namely, salt-assisted ultrasonic de-agglomeration (SAUD). Utilizing ultrasound energy and ionic salts (sodium chloride and sodium acetate), SAUD is expected to break apart thermally treated nanodiamond aggregates (~50–100 nm) and produce an aqueous slurry of de-aggregated stable colloidal nanodiamond dispersions by virtue of ionic interactions and electrostatic stabilization. Moreover, the SAUD technique neither has toxic chemicals nor is it difficult to remove impurities and therefore the isolated nanodiamonds produced are exceptionally suited for engineered nanocarbon for mechanical (composites, lubricants) and biomedical (bio-labeling, biosensing, bioimaging, theranostic) applications. We characterized the microscopic structure using complementary techniques including transmission electron microscopy combined with selected-area electron diffraction, optical and vibrational spectroscopy. We immobilized SAUD produced NDs on boron-doped diamond electrodes to investigate fundamental electrochemical properties. They included surface potential (or Fermi energy level), carrier density and mapping electrochemical (re)activity using advanced scanning electrochemical microscopy in the presence of a redox-active probe, with the aim of understanding the surface redox chemistry and the interfacial process of isolated nanodiamond particles as opposed to aggregated and untreated nanoparticles. The experimental findings are discussed in terms of stable colloids, quantum confinement and predominantly surface effects, defect sites (sp^2^–bonded C and unsaturated bonds), inner core (sp^3^–bonded C)/outer shell (sp^2^–bonded C) structure, and surface functionality. Moreover, the surface electronic states give rise to midgap states which serve as electron donors (or acceptors) depending upon the bonding (or antibonding). These are important as electroanalytical platforms for various electrocatalytic processes.

## 1. Introduction

The outstanding physical (mechanical, electrical, room temperature optical luminescence), chemical and biological (inertness, electrochemical, biocompatibility) properties make diamond an attractive material for scientists and engineers alike. While diamond is a wide bandgap semiconductor, doping with boron introduces impurity bands within an energy gap such that beyond a certain concentration this leads to semi-metallic or metallic (i.e., Mott insulator–metal transition occurs at boron concentration of ~2 × 10^20^ cm^−3^) [1] behavior. To exploit the outstanding bulk properties of diamond in the nanoworld, the size of the diamond particle is tuned and the surface can be engineered to target specific technological applications. Undoped nanodiamonds synthesized using chemical vapor deposition (CVD) are of two types i.e., nanocrystalline, NCD and ultrananocrystalline, UNCD, which are favorable for nanoelectromechanical devices and electroanalytical chemistry among other applications. Another significant form of nanodiamond, known as detonation or explosive nanodiamond (ND, hereon), is produced from detonation of carbon precursors such as trinitrotoluene (TNT) and hexogen (RDX) in oxygen-deficient conditions. The commercially available detonation nanodiamond has narrow size distribution (2–10 nm) that can be purified via air oxidation or treatment with mineral acids [2,3]. The interest in detonation nanodiamonds stemmed from their belonging to the novel nanocarbon family, specifically, the fullerenes, onion-like carbon, and nanographite [4]. A less intriguing finding was the discovery of nanodiamond in meteorites with average size 4 nm. Theoretical models (‘bucky-diamond’ versus unstructured amorphous carbon with sp, sp^2^, and sp^3^ hybridized carbon mixtures) [5,6] proposed that the individual ND particle core consists of crystalline phase diamond (sp^3^–bonded carbon; sp^3^ C) with trace nitrogen substitutional impurity into the lattice which is the source of intrinsic room temperature photoluminescence. It is surrounded by an ultrathin shell (width ca. 0.6 nm) composed of sp^2^–bonded carbon (sp^2^ C), unsaturated bonds as defects, and dangling bonds which offer rich surface redox chemistry. Additionally, in most case ND is in a highly oxidized state as a consequence of the air purification procedure resulting in surface termination with hydroxyl (–O–H), carboxylic –C(=O)–O–H, carbonyl (–C=O), lactone or ketone (R–O–R’), and ether (–C–O–C) functional groups with the associated charged state (usually amphoteric), useful for electrochemical (re)activity [7,8]. There are additional surface groups such as phenols, pyrones/chromenes, and sulfonic acids in as-synthesized material, which is not surprising as they are commonly found on graphitic or sp^2^ C surfaces.

Detonation diamond nanoparticles in the dry powder state typically form aggregates of 50–100 nm dimensions due to van der Waals forces and it is challenging to separate them as isolated nanoparticles using ball milling, power ultrasound etc. However, occasionally *s*omewhat distinguishable individual particles within aggregates of 10 nm are visible in transmission electron microscopy(TEM) [9]. Since the nanodiamond particles are proposed for preparing hard coating nanocomposites, label-free biosensing, drug delivery, electro-/biocatalysis, and quantum information technologies [7,8], the agglomerated or coalesced form poses significant limitations for practical use. For example, the uniform dispersion and interfacing (with larger density of interphases) of nanodiamond particles as reinforced agents (smaller size) with other materials such as polymers or metals for synthesizing viable nanocomposites, single-molecule bio-labeling (purity), electrocatalysis, and photon generation are some of the greatest challenges [10,11]. Conversely the large aggregation prevents any remarkable redox electrochemical activity by inhibiting the exposure and accessibility of nanodiamond rich surface chemistry [12]. While, recent progress has been made towards dispersion of detonation nanodiamond along with control of purity and surface chemistry using traditional techniques, they are not effective for ND [13,14,15]. Therefore, specific de-aggregation methods have been listed and proposed [16].

Among the known de-aggregation methods developed, those using ZrO_2_ microbead-assisted wet milling and bead-assisted sonic disintegration are the most common, producing single-digit nanodiamond particles. The process is initiated by energy of cavitation following collision and crushing and the particles can be used in various applications. However, the de-aggregation method for ND should be facile, scalable, less time-consuming, and be able to produce single-digit nanoparticles with higher crystalline phase purity. Subsequently, the methods where size of nanodiamond and microbeads become comparable during milling are desirable for efficient production of stable colloidal dispersions. Sodium chloride (NaCl) and sodium acetate (CH_3_COONa) salt crystals can provide sufficient balance of brittleness and hardness to destroy ND agglutinates as was proposed in Reference 16, while maintaining comparable size of milling bodies (NaCl and CH_3_COONa) and milled materials (ND), unlike hard uncrushable zirconia beads. Water solubility of salts also allows simple processing and complete elution from the ND producing pure colloidal dispersions. The use of salts as milling agents beyond their solubility limit operated with an ultrasound energy powered wet process is known as salt-assisted ultrasonicated de-agglomeration (SAUD, hereon) [16]. It produces nanodiamond slurry followed by centrifugation, multiple washing, and isolation of the nanodiamond particles (see experimental section for details).

B-doped diamond (BDD) films on silicon substrates provide superior chemical stability, low background currents, biocompatibility, and wider electrochemical potential window sensing electrodes [17,18,19,20,21,22,23,24] allowing higher sensitivity and lower detection limits of analytes and suitable immobilization platforms for nanomaterials or biomolecules attractive for electroanalytical experiments [25,26,27,28,29,30,31,32]. In view of their rich electrocatalytic as well as biocatalytic properties, recent literature explored the properties of ND colloid suspensions as collection agents for pre-concentration of proteins or DNA oligomers, fluorescent imaging in living cells [17,18,19,20] and drug delivery [21,22,23]. Other groups examined the surface groups from as-synthesized oxidized and fluorinated ND [24,25,26,27]. Motivated by the literature reports, the electrochemical properties of SAUD processed ND particle are here studied. As proposed, the surface of undoped nanodiamond gives rise to surface electronic states within the bandgap or midgap states. These surface states elucidates both an excess of unpaired electrons (electron donors) and unfilled electronic states (acceptors) and support catalytic redox processes in the presence of redox-active molecules via a feedback mechanism [25], revisited in Reference [12]. They are usually related to tunneling of valence band electrons into the lowest unoccupied electronic levels of an adsorbed layer of electrolyte when immobilized on heavily doped BDD films [33,34,35,36,37,38,39,40,41]. To this end, the focus of this work is multifold including (1) to work out the surface redox chemistry of SAUD processed isolated nanodiamond particles; (2) to determine surface electronic properties (conductivity, potential, transfer doping, charge density) and (3) to gain insights into quantify surface defects distribution, diffusion coefficient and heterogeneous rate constants using advanced scanning electrochemical microscopy. The experimental findings emphasize the interplay of size effects and inside atoms, diamond core/uncoordinated or unsaturated surface atoms (outer shell), both predominating mainly surface atoms in enhancing the electrochemical redox properties and electroanalytical properties [12,25,26].

## 2. Experimental

### 2.1. Materials and Methods

Salt-assisted ultrasonication de-aggregation and immobilization of nanodiamond on B-doped diamond electrodes.

Two different detonation nanodiamonds were acquired [Alit Co., Kiev, Ukraine provided by V. Padalko; ND1 and International Technology Center, Adamas Nanotechnologies, Raleigh, NC, USA; ND2] for testing the adapted SAUD method. Their individual average size ranged as ND1 (5–10 nm) and ND2 (3–5 nm) which were confirmed using high-resolution transmission electron microscopy provided below. As-received ND samples were thermally treated in a Carbolite box furnace at ~450 °C in air for 2 h to eliminate graphitic carbon and to optimize site density of oxygenated surface functional moieties such as carboxylated (–COOH) groups [42]. These thermally treated air oxidized NDs contain >95 wt.% diamond phase terminated with –COOH groups, labeled as ND1–COOH and ND2–COOH indicating dominant surface functional groups.

ND powder (500 mg) and NaCl (20 mg) salt were mixed for 20 min. in an Agate mortar and pestle and placed into a 40 mL glass vial along with 10 mL distilled water. The prepared mixture was sonicated using an ultrasonication bath (Model Branson, Fisher Scientific, Peabody, MA, USA) for 2 h at 70% output power and 50 kHz frequency. After the ultrasonication, the dispersions were split into half and put it into 50 mL plastic falcon centrifuge tubes and dispersed in distilled water up to 50 mL. Each of these samples was centrifuged using an Eppendorf centrifuge (Sorvall Lynx Model 4000) at 4000 rpm at room temperature for 15 min. and the clear supernatant was discarded. Both of the nanodiamond sample precipitates were re-dispersed in distilled water (50 mL each) and centrifuged again but at 10,000 rpm for 50 min. at room temperature. Once again the clear supernatant was discarded and the wet nanodiamond precipitates were re-dispersed and this time in 5 mL each for structural and optical characterization. A standard AgNO_3_ assay was used to demonstrate the removal of Cl^−^ ions in the SAUD samples which were labeled as (ND1–COOH–SAUD)_NaCl_ and (ND2–COOH–SAUD)_NaCl_. These samples were washed with distilled water thrice as described above and the water let to evaporate from the samples in air at room temperature for 48–72 h resulting in dark gray solid ND “flakes” with 65–70 wt % yield of the initial ND mass. A similar procedure was followed for sodium acetate salt (NaAc) labelled as (ND1–COOH–SAUD)_NaAc_ and (ND2–COOH–SAUD)_NaAc_. These collidal dispersions were drop-casted on TEM grids and immobilized as overlayers onto clean BDD conductive substrates as electrochemical electrodes using Eppendorf micro-pipetting (~3–5 μL) for microstructural and electrochemical properties, respectively. The BDD substrates were purchased commercially (Fraunhofer USA-CCD, East Lansing, MI, USA) and they were synthesized using MWCVD (Micrwave chemical vapor deposition) technique described elsewhere [12]. The BDD electrodes were cleaned by mechanical polishing with an alumina suspension, 0.05 μm (Buehler, Lake Buff, IL, USA) and rinsed with deionized water using microcloth polishing pads (Buehler, Lake Buff, IL, USA). These ND modified BDD electrodes were air dried for 2 h and checked under an optical microscope for surface homogeneity.

All the chemicals were purchased from Sigma-Aldrich (St. Louis, MO, USA) and Fisher Scientific (Nashville, TN, USA) including sodium chloride (NaCl, >99%), sodium acetate (CH_3_COONa, >99%), potassium sulfate (K_2_SO_4_, >99.5%), ferrocene methanol (FcMeOH, >99%) and used without further purification. The dispersion was prepared using Milli-Q water with resistivity >18.2 MΩ cm (Millipore Sigma, Billerica, MA, USA). All the values reported in this work are an average of three samples to illustrate reproducibility.

### 2.2. Structural and Spectroscopic Characterization

Samples were characterized to obtain surface morphology, micro-/nanoscale structure, optical (electronic) and Raman (lattice vibration) spectral signatures. Using a micro-syringe, a few drops of SAUD processed colloidal suspensions and unprocessed dispersions were coated as overlayer on commercial silicon and boron-doped diamond thin film electrodes for Raman spectroscopy. For transmission electron microscopy, a few drops were distributed onto commercial lacey carbon coated Cu grids (Ted Pella, Inc., Redding, CA, USA) and air dried. TEM images were taken using a JEOL instrument (Model 1400 Plus, Peabody, MA, USA) operating in cryo-EM and SAED modes at 100 kV and 1 nA from a LaB_6_ gun with a Be specimen holder, a Gresham SiLi detector with Moxtek AP3.3 window and with an AMT 8 Mpixel cooled camera. TEM measurements helped to determine the nanoparticle size distribution. For SAED patterns, we used 0.10 μm aperture producing a small spot size and spread the beam to increase the electron coherence length at the samples. For optical properties, we measured UV–Vis absorbance spectra for all the colloidal dispersions using aBioTek spectrometer (Model Synergy H1 Multi-mode plate Reader, Winooski, VT, USA) equipped with a xenon lamp as broadband excitation source of wavelength ranging from 400–800 nm in intervals of 1 nm. The mass extinction coefficient was determined from the absorption plots. For fluorescence emission measurements, colloidal dispersions were placed in a 96 vial plate reader with ~500 μL each (6 mm width). The excitation wavelength used was λex = 370 nm (and 530 nm) and the spectra were measured between 350 and 550 nm (from 600 to 680 nm) with a wavelength interval 1 nm (and 0.5 nm) at room temperature. We also determined the concentration (or molar absorptivity) of SAUD processed nanodiamond at 600 nm and they were determined to be 2.5 wt %, similar for SAUD processed NDs. Raman spectra were measured to determine carbon bonding phases. The Raman spectra were recorded using a micro-Raman spectrometer (Model InVia Renishaw *plc*, Gloucestershire, UK) equipped with laser providing excitation wavelength 633 nm (E_L_ = 1.92 eV) and ~1 mW or less incident at the sample. The reflected light was filtered using an edge filter to remove the laser excitation cutting at ~100 cm^−1^ and sent to the spectrometer. The scattered light from the sample was collected in backscattering geometry transmitted and detected by a CCD camera. An objective lens of 50× was used providing a spot size of ~1–2 μm and the laser power on the sample was maintained between <0.1–0.5 mW (or 1% or 5%) using neutral density filters to avoid local heating effects preventing photo-thermal degradation. The Raman spectra were acquired from 60 s to 100 s depending upon the laser power used and to maximize throughput signal. Raman shifts ranged from 1000 cm^−1^ to 2000 cm^−1^ with spectral resolution of 1 cm^−1^.

### 2.3. Electrochemical Properties and Scanning Electrochemical Microscopy

The electrochemical properties were carried out using a bipotentiostat electrochemical workstation and Scanning Electrochemical Microscope (Model 920D CH Instruments, Inc., Austin, TX, USA) equipped with CHI (ver. 12.03) software. The measurements were performed in a cusom-built three-electrode electrochemical cell. The working electrode (WE) is ND modified BDD electrodes of size 4 × 1 cm^2^ and BDD as control with 3 mm diameter Pt wire counter electrode (CE) and a reference electrode (RE), where a reference potential was achieved using Ag/AgCl (saturated 3 M KCl) electrode potential. The base electrolyte used is a mixturesof 0.05 M K_2_SO_4_ (pH 7.02) with 10 μM of FcMeOH as redox probe. All the samples were electrochemically pre-processed at scan rate 5 mV/s a few times to clean the surface debris and to ease the noise from the cyclic voltammetry (CV) scans. The electrochemical characterization included CV at scan rates 10, 20, 50, 100, and 500 mV/s in the potential window +0.6 V to −0.2 V. Electrochemical impedance spectroscopy technique was carried out with 0.05 M K_2_SO_4_ in the potential range +0.6 V to −0.2 V with step size 10 mV and the spectra were measured at various frequencies 50, 100, 250, 500, 1000, and 5000 Hz with ac amplitude 10 mV. Before impedance spectra registration, the electrode was conditioned at the fixed potential for 5–10 min. Prior to electrochemical tests the electrolytes were purged with Ar for 50 min.

**Figure 1:**
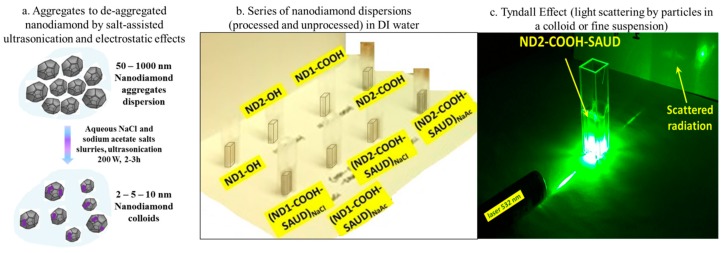
(**a**) Scheme of de-aggregation of nanodiamond following salt-assisted ultrasonication. The salts used are sodium chloride (NaCl) and sodium acetate (CH_3_COONa); (**b**) The series of nanodiamond dispersions and (**c**) Tyndall effect—light scattering by a representative de-aggregated nanodiamond (ND2-COOH-SAUD) colloid.

For scanning electrochemical microscopy (SECM, Model 920D CH Instruments, Inc., Austin, TX, USA), the working electrode tip is a 5–10 μm diameter Pt wire sealed in glass with RG ≅ 3 (R is the radius of tip and G is the diameter of glass) and the other working electrode is a ND-modified BDD electrodes. The electrodes were clamped into the bottom of a Teflon cell by means of an O-ring assembly so that the SECM tip could approach from above to measure the probe approach curves in negative feedback mode. The bias voltage on ND surfaces is controlled by means of Cu wire connection prepared using silver epoxy and dried under lamp heating (~80 °C for 1 h). All the CV measurements in SECM are measured with 0.05 M K_2_SO_4_ base electrolyte and redox probe 5 mM FcMeOH at scan rate 20 mV/s. To measure heterogeneous electron transfer rate (k_ET_), we measured probe approach curves in support electrolyte 0.05 M K_2_SO_4_ with redox probe 5 mM FcMeOH that has a standard potential E° = +0.21 V versus Ag/AgCl similar to potassium ferricyanide, K_3_Fe(CN)_6_. For SECM imaging, both the electrodes (tip and sample) were biased such that V_t_ = +0.25 V and V_S_ = −0.4 V to ensure complete oxidation of Fe(II) species generated at the tip originally present in the electrolyte solution to Fe(III) thus establishing a redox cycle. The tip was rastered over the working electrode surface area (250 μm × 250 μm) at a constant tip-substrate separation lying between ≤10 and 30 μm to generate a feedback image with approximate resolution 1 μm and sub-nanoampere tip current (*i*_T_) sensitivity. The probe approach curves were fitted following empirical equations provided in reference [12] and SECM two and three-dimensional “heat” maps were generated using Origin software (ver. 16.0, OriginLab, Northampton, MA, USA). Figure 1 shows the schematic diagram of the SAUD processing steps (left panel), illustrates the resulting colloidal dispersion (middle panel) state of SAUD processed NDs nanoparticles as well as the Tyndall Effect (light scattering by particles in colloid or fine suspension, right panel).

## 3. Results and Discussion

### 3.1. Microstructural Properties

**Figure 2:**
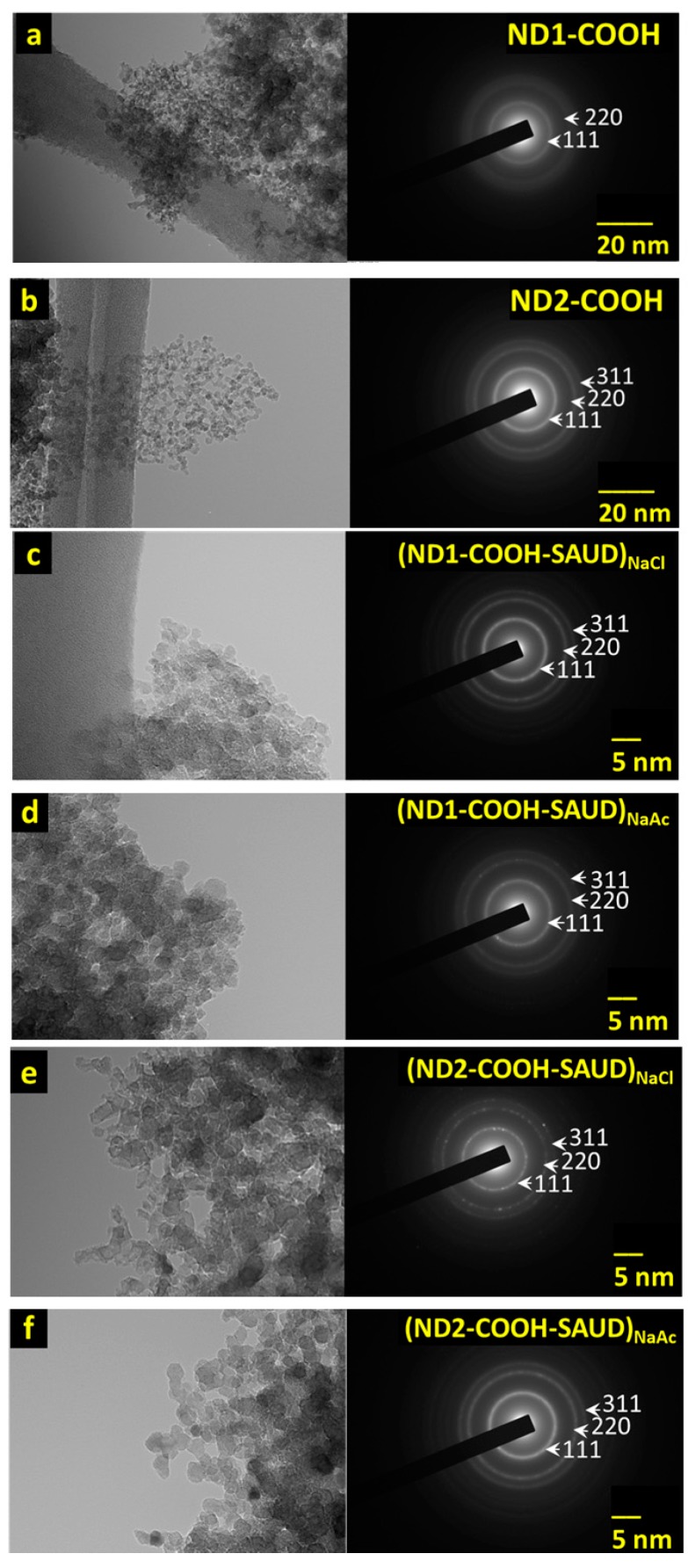
(**a**–**f**) Transmission electron microscopy (TEM) micrographs of unprocessed and two different salts (sodium chloride; NaCl and sodium acetate; NaAc) processed nanodiamond series (ND1-COOH and ND2-COOH) and corresponding selected area electron diffraction showing characteristic diamond peaks with (hkl) assignment. The scale bar is 20 nm for the micrographs and selected-area electron diffraction (SAED) images are shown at the bottom of the images.

**Figure 3:**
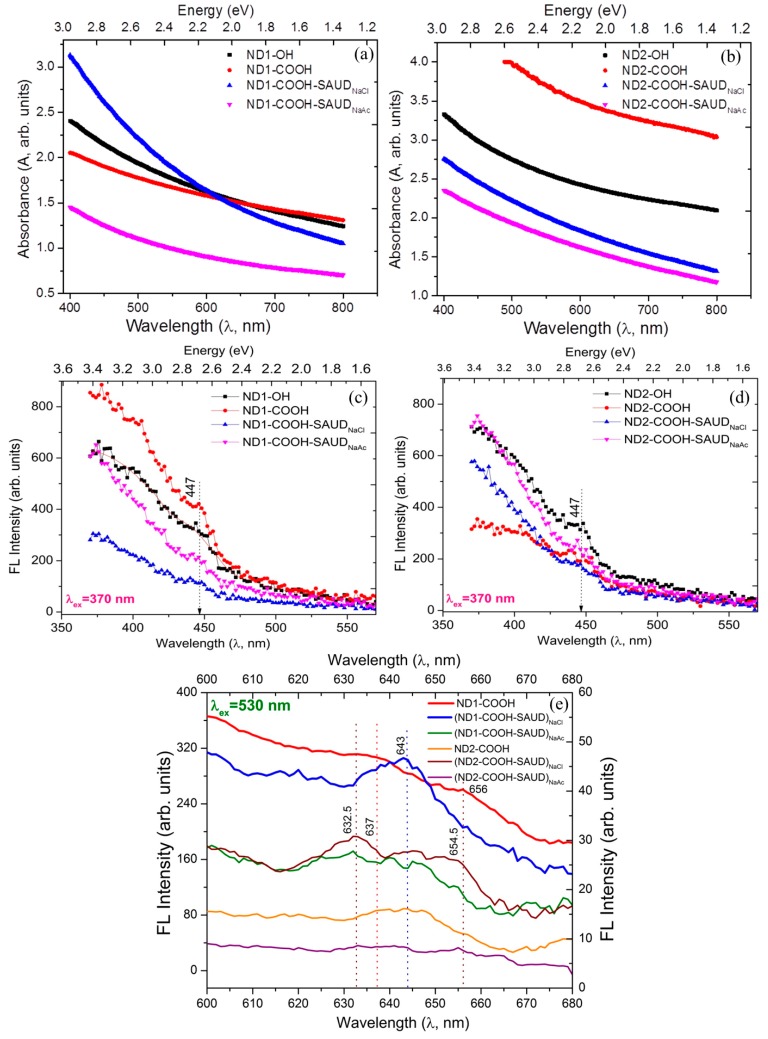
(**a**,**b**) UV-Visible optical absorption and (**c**–**e**) fluorescence spectra excited at *λ*_L_ = 370 nm and 530 nm of unprocessed and salt-processed nanodiamond series (ND1-COOH and ND2-COOH).

The as-received NDs characterization reported elsewhere [12] show aggregates with mean size of 100–300 nm. Figure 2 shows transmission electron microscopy (TEM) images of air oxidized (panel a,b) and SAUD processed (panel c–f) NDs. The samples are composed of relatively uniform dispersed spherical particulates without dense agglomeration, which becomes much more apparent and discrete for SAUD processed ND particles, especially ND2 samples with mean diameter 5 nm or less. Also, provided are the selected-area electron diffraction (SAED) patterns confirming the SAUD processing which retained a crystalline diamond structure. The SAED rings correspond to (111), (220), and to (311) NDs Miller planes with interplanar spacing d_hkl_ = 0.203, 0.127, and 0.110 nm, respectively. The measured interplanar spacing matches those of a crystal structure of a face-centered cubic (FCC) diamond phase. The elemental composition and mapping were assessed using energy dispersive X-ray spectroscopy (EDX) which shows that the samples are primarily carbon (C) with residual oxygen (O) and occasionally sodium (Na) from milling salt agents depending upon the probe area indicative of high phase purity. The signals from other elements are also observed in EDX spectra occurring from the TEM grid (Cu) and TEM detector (Si) with no other contaminants (such as SiO_2_, Cl, etc.). SAUD processed ND suspensions result in color changes in contrast to those observed for unprocessed, as-received or air oxidized ND dispersions. It is well-known that well-dispersed NDs in water have a darker color compared to the aggregated samples (light gray), the origin of which is debatable. However, by adjusting the pH one can change the color during de-aggregation, which is not related to the light absorbance by graphitic carbon impurities. There are factors including light scattering (see Figure 1c, Tyndall Effect), absorbance by surface states, and others contributing to the colored state of <10 nm size ND colloid suspensions in addition to bulk absorbance [43,44]. Light absorbance can be used to determine the ND concentration quantitatively (or molar absorptivity) which is carried out at 500 nm for ND samples (as-received, air oxidized, unprocessed, and SAUD processed). Figure 3a,b presents a typical view of absorption spectra for air oxidized and SAUD processed NDs in the UV–Vis range. As shown in Figure 3, the absorption spectra have no specific absorption bands and appear as smooth curves, which grow exponentially while the wavelength is shifted toward the UV region. In general, such behavior of spectra is normal for weakly absorbing but strongly scattering samples. By virtue of an additional investigation, we attempted to evaluate the contributions of light absorption and scattering from the two sets of the nanodiamond (ND1 and ND2) series. ND1 is characterized by lower absorptivity (~2.5) as compared with ND2 absorptivity (~4.0). We presume that such differences in the spectra correspond to a different size of particles in solution and this hypothesis was elucidated by measurements of the size of the aggregates in solution by dynamic light scattering experiments (see Figure S1, Supplementary Materials). We also checked the stability of the absorption spectra and found that the absorbance changed marginally and it increased by 0.01–0.05 in the UV region after a few days of preparation for both the ND1 and ND2 solutions and returned to the initial state after ultrasonic treatment for 1 h. Using Lambert-Beer’s Law, the molar concentration values determined from the absorption spectra are 0.92 (1.89), 1.35 (1.21), and 0.82 (0.74) mg·mL^−1^·cm^−1^ for ND1 (ND2) series following, *ε* = A/*lc*, where *l* is width of cell, *c* is the concentration (mg/mL), and A is the absorbance. The minimal particle size achieved with SAUD is ≤10 nm with a small fraction of ≥10 nm particles size distribution measured using dynamic light scattering, DLS (Figure S1, Supplementary Materials). At the same time the correlation function or more precisely the power spectral density (PSD) of NDs, regardless of surface chemistry show the presence of larger agglomerates in the 100 nm range (Figure S1 red curves, Supplementary Materials). These PSDs of particle size distribution were measured with 2.5 wt % NDs. Nevertheless, qualitative conclusions can be drawn about the particle size of NDs in the dispersions. SAUD processed NDs were dried and re-dispersed into colloidal suspensions using mild ultrasonication, which is significantly advantageous as compared with NDs processed using other de-aggregation techniques resulting in larger agglomerates [12,15]. Sodium chloride and sodium acetate salts play a vital role as mild milling agents beyond the solubility limit.

For instance, apart from crushing ND agglomerates, NaCl provides Na^+^ ions which can form sodium salts with carboxyl (–COOH) groups on the surface of ND particles. This hypothesis is partially corroborated with the help of EDX methods measuring surface elemental composition which showed the occasional presence of Na. Also, the pH of SAUD ND colloids have a slight increase in pH (7.81 → 9.92) which may be due to dissociation of the sodium salts and of weak acids i.e., –COOH groups on the NDs may be forming into a strong base (i.e., NaOH). Interestingly, SAUD processing is applicable to other salts such as KCl and other metal salts including chlorides, sulfates or nitrates of Cu, Fe, Ni, Co. In fact, the presence of metals Na, K resulting in single-digit SAUD NDs can be beneficial for single-molecule biomedical/bioimaging since they have been shown to inhibit proliferation of HeLa cancer cells [45,46,47] and metal-matrix composites. Furthermore, to produce single-digit NDs colloids with no metal salts, we would resort to organic crystalline compounds (sucrose, phenols, and quinones, etc.). Therefore the efficiency of SAUD is usually studied with non-aqueous organic solvents such as THF (tetrahydrofuran), chloroform, NMP (*N*-methylpyrrolidone), DMF (dimethyformamide), etc. for polymer-ND nanocomposites. Figure 3 shows photoluminescence (PL) emission at two different excitation wavelengths (370 nm and 530 nm) displaying emission peaks at ~447 nm, ~632 nm, and ~640 nm, respectively. It is well-known that the origin of PL emission is due to defects and admixtures in the ND particle core formed by a crystalline diamond lattice. Different fluorescence emission peaks largely depend upon the different methods of production; size effects and different wavelengths optically excite different defects. Most importantly, ND fluorescence is immune to photobleaching and therefore nanodiamond particles may become useful in developing ND-based label free biomarkers for life sciences [17,48,49]. Under green wavelength (530 nm) illumination, NDs showed clear nitrogen-vacancy PL with neutral nitrogen-vacancy (N-V)° and negatively charged nitrogen-vacancy (N-V)^−^ centers of which the zero-phonon lines peaked at ~575 nm and ~638 nm, respectively (see Figure 3, panels c–e).

Other than the core, surface moieties and the dielectric environment equally play an important role in PL properties [49]. All SAUD processed ND colloidal solutions produced are sufficiently stable showing no precipitation when stored at ambient condition for more than 5–6 months with mean particle size of 4–9 nm. Important to note is that by varying the concentration of NDs, the mean size can be varied from smaller to larger which hypothetically has some physical origin arising due to interparticle distance equilibrium beyond which the NDs do not agglomerate. An increase in interparticle distance between their nearest neighbors in the colloidal state results in potential energy gain. This energy penalty prevents formation of aggregation and stabilizes the single-particle colloidal state. Any deviation from this state will decrease this pair attraction triggering agglomeration, which requires both theoretical (e.g., MD simulations) calculations and experimental corroborations. To this end, we attempted to study the surface properties of isolated nanosized ND particles using traditional and advanced electrochemistry.

**Figure 4:**
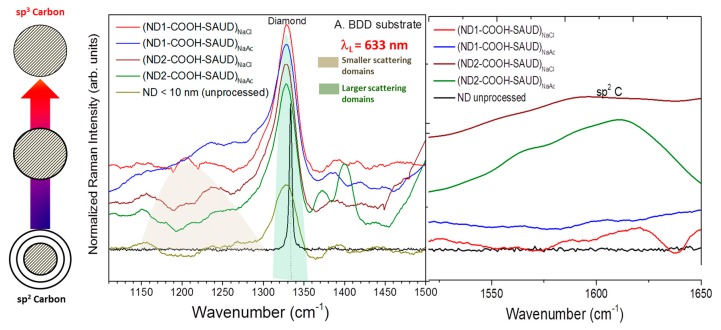
Normalized Raman spectra of nanodiamond series (ND1-COOH and ND2-COOH) displaying characteristic diamond Raman peaks along with micro-diamond peak and the non-diamond region.

Raman spectroscopy is an important analytical tool to gather information about molecular and crystal lattice vibrations. It is used to characterize carbon-based materials since it is sensitive to different types of carbon–carbon bonding, polymorphism, and is capable of monitoring changes in Raman bands when the size of the crystals is decreased to nanoscale [50,51,52]. Figure 4 shows the Raman spectra of polycrystalline diamond as reference and ND particulates. The polycrystalline diamond shows a characteristic diamond peak at ~1332 cm^−1^ caused by a discrete zone–center phonon, which is triply degenerate. It becomes apparent that SAUD processing does not alter the surface chemistry of air oxidized NDs and it works well for differently sourced NDs. As for SAUD processed and air oxidized NDs, they both show a characteristic first-order diamond (sp^3^ C phase) peak which is downshifted by a few wavenumbers ~1325 cm^−1^ (ca. 1332.5 cm^−1^) albeit relatively broader Г_FWHM_ = 11.4 cm^−1^ (ca. 1.1 cm^−1^) and a shoulder band at ~1250 cm^−1^, predicted for nanosize particle domains. However, the shift in the diamond peak is in the opposite direction to those assigned typically due to phonon confinement [51,52]. To this end, we attempted to provide a physical interpretation which supports the observation of red shift which is described as surface effects in which a semiconductor nanocrystal, particularly nanodiamond is composed of inside atoms (core) surrounded by surface atoms (shell). Therefore, the Raman spectral shift arises due to competing influences from under-coordinated surface atoms (i.e., surface effects) [51,52,53] and bond-length change. Raman shifts are resolved into contributions from surface and core bonds, the former is defined as atoms with an imperfect coordination number (surface dangling bonds). Therefore the size dependent Raman shift of nanodiamond is expressed as [53].

$\Delta \varpi (D)=F^{surface}\Delta \varpi {\left(D\right)}^{surface-nanocrystal}+(1-F^{surface})\Delta \varpi {\left(D\right)}^{bulk-nanocrystal}$, where F^surface^ is the fraction of surface bonds with respect to total number of bonds, $\Delta \varpi {\left(D\right)}^{surface-nanocrystal}$ is the Raman shift of a surface nanocrystal whose atomic coordination number and bond length are entirely due to surface nanocrystal and $\Delta \varpi {\left(D\right)}^{bulk-nanocrystal}$ is entirely due to bulk nanocrystal. To understand the role of surface effects, Gao et al. [53], estimated the contribution in Raman shift by the following: $\eta =|F^{surface}\Delta \varpi {\left(D\right)}^{surface}/\Delta \varpi (D)|$. The calculated values for η of 40% and 10% are for nanodiamond and for Si nanocrystals, respectively. It becomes apparent that while the larger shift in Si nanocrystals results from quantum effects, the contribution of the surface effect in nanodiamond is too large to be ignored. Following equation $\varpi (D)=1332.5-94.9QN^{-1/3}-24.5F^{surface}(cm^{-1})$ where *N* is the number of carbon atoms [53], the size of the SAUD processed nanodiamond determined is approximately 5.0–5.4 nm, corroborated with high-resolution transmission electron microscopy.

Additionally, red shift can be related to tensile or residual stress and/or thermal stress induced by focused laser radiation [54]. To elucidate the absence of photothermal-induced shifts or damage to nanodiamond samples, we changed the laser power (from 0.1 mW to 0.5 mW), cycled the laser power a few times and monitored the peak position which presented no detectable change in the Raman band position. Broad bands between ~1580 and 1710 cm^−1^ are assigned to delocalized *π* (oxidizable)–*π* (inoxidizable)* character rather than extended graphite-like sp^2^ C defects [12,26,50]. They are usually associated with alcohol and carbonyl (1624 cm^−1^ (*ν*_O=HAbs._) and 1725 cm^−1^ (*ν*_C=O_)) species, respectively, on the surface of nanodiamond particles [55]. There is no indication of D band which usually occurs at 1350 cm^−1^ and therefore it is apparent that there is minimal graphitic sp^2^ C phase. However, the convoluted set of peaks between 1100 and 1450 cm^−1^ contain a mix of bending and stretching modes of various C–C, C–H, and C–O groups from surface functionalities. These various surface functional groups are pertinent for the presence of redox active peaks of interest that include ketone or quinine-like moieties participating in redox cycling while investigating surface redox chemistry of immobilized nanodiamond overlayers [12,56].

### 3.2. Electrochemical Measurements Cyclic Voltammetry

**Figure 5:**
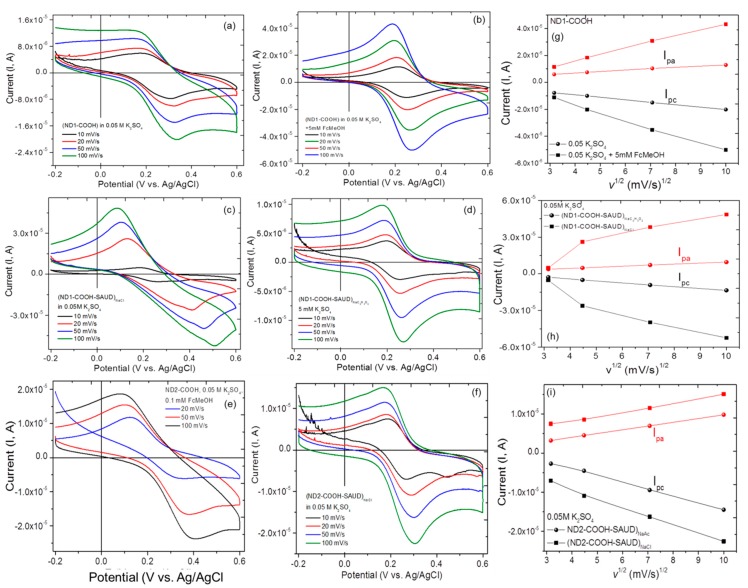
(**a**–**f**) Cyclic voltammograms of annealed nanodiamond (ND1–COOH and ND2–COOH) and two different salts (sodium chloride; NaCl and sodium acetate; NaAc) processed nanodiamond (ND1–COOH–SAUD and ND2–COOH–SAUD) in base electrolyte 0.05 M K_2_SO_4_ and with the electrochemical probe molecule ferrocenemethanol (5 mM FcMeOH) that is immobilized on BDD electrodes with scan rate. (**g**–**i**) The maximum anodic current (I_pa_) and cathodic current (I_ca_) versus scan rate displaying a linear behavior is also plotted.

This section provides cyclic voltammetry (CV), electrochemical impedance versus potential and scanning electrochemical microscopy (SECM) studies for all NDs which are immobilized on BDD thin films as working electrodes. Figure 5 shows CV curves of air oxidized (ND1-COOH, ND2-COOH) and SAUD processed NDs [(ND1-COOH-SAUD)_NaCl_, (ND1-COOH-SAUD)_NaAc_, (ND2-COOH-SAUD)_NaCl_] with varying scan rates. All of the NDs show current enhancement with increasing scan rate. Due to finite electrical resistance in ND particulates, the CV response was superimposed on a linearly sloping background albeit marginal. The redox peak separation potential $\Delta E_{p}=|E_{p}^{ox}-E_{p}^{red}|$ is ~107 mV at the nanodiamond-modified electrodes, which is larger than the theoretically predicted value of 59 mV for a diffusion-controlled one electron transfer reversible redox reaction. In our previous reports we systematically elucidated the role of diamond particle size and scan rate in the current enhancement for a redox peak by five-fold compared to those of microcrystalline diamond [12]. The electrochemical (re)activity enhancement of undoped nanodiamond particles was attributed to the density of surface functional groups as well as outer graphitic shell defect sites, which can vary with air-oxidation treatment thus exposing the crystalline facets of diamond core and edge plane sites as redox active sites. The oxidation takes place through electron exchange between the ND surface and the redox probe which are reduced concomitantly with the generation of FcMeOH^+^ i.e., FcMeOH + ND_OX_ → FcMeOH^+^ + ND_red_, which corroborates with our previously reported observations. However, the process occurred with much ease without requiring surface cleaning or multiple repeated CV cycling [12,25,26]. Interestingly, we did not observe two oxidation waves under the experimental conditions employed in any of the ND samples studied, i.e., main peak (diffusion-controlled) and pre-peak to reduction wave as reported [26]. We found merged peak behavior, the position of which is midway between the weak pre-peak and main peak for (ND1–COOH–SAUD)_NaCl_, ND2–COOH and (ND2–COOH–SAUD)_NaAc_ samples. From our previous work [12] and the literature [26], it was reported that with decreased ionic strength of electrolyte, a pre-peak emerges between 0.1 V and 0.2 V in the oxidation wave and the corresponding reduction wave appears between 0.2 and 0.4 V. This behavior is indicative of outer-sphere adsorption of oxidation species at the electrode surface [12,57,58,59] Accordingly the Debye-Hückel screening length to which the electric field of a surface can be felt is inversely proportional to the electrolyte concentration. Alternatively, with decreasing electrolyte concentration the core-shell interface of nanodiamond particles is accessible for gaining insight into diamond (sp^3^ C)-graphite-like (sp^2^ C) bonding interphases. Therefore, the unsaturated carbon bonding and surface functionalities allow hydrophobic and *π*(oxidized)-π*(reduced) stacking interactions with aromatic pentacyclo–moieties of the FcMeOH molecule. Also, the neutral FcMeOH is more likely to be adjacent to the SAUD processed nanodiamond (NDs), which may allow tunneling of FcMeOH^+^ ions due to electrostatic repulsion with negatively charged SAUD NDs much more effectively than if they were in the aggregated state. In order to determine the diffusion coefficient (D), the heterogeneous rate transfer constant (k_ET_), and the effective adsorption area from surface redox electrochemical properties, we carried out quantitative analysis and calculations provided in the subsection below. Presumably, the adsorbed FcMeOH^+^ regeneration rate by reduction at the ND surface is fast enough (smaller nanodiamond), the limit to this reaction is simply governed by how much FcMeOH^+^ is adsorbed at the ND surface. Moreover, redox confined species and the corresponding cathodic (*i*_pc_) and anodic (*i*_pa_) peak currents are almost linearly dependent upon the scan rates (see Figure 5g–i), reminiscent of convoluted diffusion-limited (mass transport) and surface confined charge adsorption behavior. The magnitude of the current observed is typically governed by the Randles-Ševćik equation for the quasi-reversible transfer process following: $I_{rev}=0.446FAC{\left(\frac{FD\nu }{RT}\right)}^{0.5}$ (or $I_{rev}=\left(2.69*10^{5}\right)n^{3/2}ACD^{1/2}v^{1/2}$), where *A* is the geometric area of the electrode (cm^2^), *F* is the Faraday Constant (C mol^−1^), *D* is the diffusion coefficient (cm^2^ s^−1^), *C* is the concentration (mol cm^−3^), *v* is the scan rate (V/s), *R* and T are usual constants, and *n* (=1) is the total number of electrons transferred in the electrochemical process [59,60]. Likewise, for surface confined adsorption and redox species, the current varies as follows: $I_{rev}=\frac{n^{2}F^{2}}{4RT}\nu AF\Gamma$_R_, where A the area under the peak is given by nAFΓ_R_ and ΔE_1/2_ = 90.6/n mV, almost lying at the borderline of Nernstian and quasi-reversible electron transfer behavior. For quasi-reversible systems, the peaks appear asymmetric and the redox peak potentials separate from each other. The analysis of the current helped to determine D that ranged between 4.5 × 10^−9^ and 7 × 10^−10^ m^2^ s^−1^ for ND2–COOH–SAUD, ND2–COOH–SAUD, ND2–COOH and ND1–COOH in decreasing order, which further reduces for as-received ND (ND2–OH and ND1–OH) samples. While the adsorption-mediated process is observed at slower scan rates due to the lower flux of FcMeOH and therefore the mass transport of reactant can keep up with the rate of regeneration of the available FcMeOH^+^ at faster scan rates, the higher flux FcMeOH leads to diffusion-controlled currents overwhelming the adsorption response. Therefore, under these conditions, a steady-state current (*i_ss_*) begins to emerge. Also with FcMeOH^+^ species build up, there are insufficient nanodiamond surface functionalities to maintain the catalytic cycle leading to a steady-state current state. The effective electrode area (*A_eff_*) is related to *i*_ss_ following: ${\left(\frac{i_{ss}}{4nFDC}\right)}^{2}$ resulting in *D* = 5 × 10^−9^ m^2^ s^−1^ for the ND2–COOH–SAUD_NaCl_ sample. From the experimental *i_ss_* (0.97 nA for FcMeOH) [12,60], we calculated *A*_eff_ of SAUD NDs to be ~98 ± 12 μm^2^ for all samples. It is also critical to determine the effective heterogeneous rate transfer constant (*k_ET_*). We estimated the apparent rate constant at the ND surfaces with the SECM probe approach curves discussed in the subsection below.

#### 3.2.1. AC Impedance versus Potential

**Figure 6:**
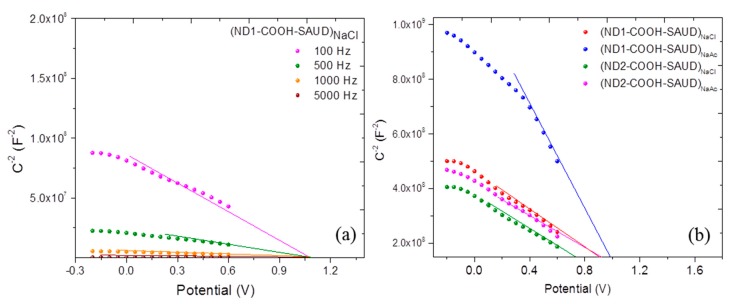
(**a**) Mott-Schottky plots for ND1–COOH–SAUD sample for different frequencies; (**b**) Mott-Schottky diagrams for all the salt-processed (sodium chloride; NaCl and sodium acetate; NaAc) nanodiamond ND1–COOH–SAUD, ND2–COOH–SAUD samples.

In the potential range where any Faradaic process occurs with the electrode immersed in 0.05 M K_2_SO_4_, the impedance spectra between 100 and 5000 Hz were measured. The selected potential range covers the regime from +0.6 to −0.2 V (see Figure 6). The spectra collected serve as the input for Mott-Schottky behaviour which is applied frequently for the electrochemical characterization of semiconducting material surfaces including nanocarbons [61,62]. This helps to determine carrier densities (N) and the flatband or Fermi potential energy (*E_f_*). The analysis is based on such that the capacitance of the space charge layer (*C_sc_*) is anticipated to be significantly lower compared to the outer sphere Helmholtz layer and depends on the type of semiconductor n (or p) [63]: $\;\frac{1}{\;C_{s}^{2}}=\frac{2}{e\varepsilon \varepsilon oN}\left(\pm E-E_{f}-\frac{kT}{e}\right)$, where *ε* is the dielectric constant of the semiconductor, *ε*_0_ is the permittivity of free space (8.85 × 10^−14^ F/cm), *e* is the electron charge (1.602 × 10^−19^ C), N is the donor (or acceptor) carrier densities (/cm^3^), E is the applied potential, *E_f_* is the flatband potential, *k* is the Boltzmann constant, and T the absolute temperature. From the 1/*C_sc_*^2^ versus applied potential plots with varying frequency, the carrier density (*N*) and Fermi level energy (*E_f_*) are determined from the slope and intercept extrapolation where 1/*C_sc_*^2^ = 0, respectively. Measurements of *E_f_* can provide useful information about the charge types associated with the surface of the ND particles. Moreover, the type of semiconductor is identified from the slope of Mott-Schottky plots, i.e., positive slope implies n-type whereas negative slope is typical for p-type carrier conduction. The analysis of impedance spectra was performed where *C_sc_* resulted from using C = −1/(2*πf*Z″) where Z″ is the imaginary part of impedance for a selected frequency [62]. On the basis of the results presented in Figure 6 for SAUD processed NDs, where Mott-Schottky plots for different frequencies covering both high (5000 Hz) and low frequency (100 Hz) regions are shown, we noted no frequency dispersion usually attributed to dielectric relaxation in the depletion layer or deep donor levels [63,64]. The values of *E_f_* together with N_A_ are enlisted in Table 1. For carrier density calculations, the dielectric constant of 5.7 (same as single–crystal diamond) was used. All the Mott-Schottky plots exhibited a distinct negative slope indicative of p-type carrier conduction [63,64,65,66,67,68,69]. Since such data for nanodiamond overlayers immobilized on BDD have not yet been reported, the values and the trend in comparison to other carbon-based materials are challenging. The shifts in *E_f_* indicate that the ND surfaces play a vital role which strongly affects the interfacial electron energetics while ensuring electrostatic stabilization of the colloidal dispersions produced by the SAUD process. Knowing that the flatband potential of a p-type semiconductor is located near the vicinity of a valence band [66,67,68,69], it is suggestive that the whole band offset, namely, the location of conduction and valence bands could be modified [66]. The estimated *N* values are higher compared to the ones reported for typical boron doped diamond [66,67] and could result mainly from surface chemical composition and the core-shell carbon phases (sp^2^ C/sp^3^ C ratio).

#### 3.2.2. Scanning Electrochemical Microscopy (SECM)

**Figure 7:**
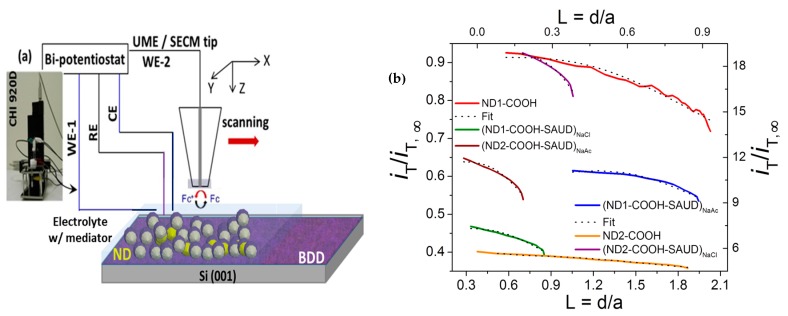
(**a**) scanning electrochemical microscopy (SECM) with (ultra) microelectrode (UME) tip as working (WE), counter (CE) and reference (RE) electrodes operating in feedback mode during oxidation of Redox (R/O) mediator species in base electrolyte at the tip positioned for ND immobilized BDD electrode substrate; (**b**) Probe approach curves for all the unprocessed and processed nanodiamond samples indicative of extent of semiconducting (or insulating) behavior at the solid/liquid interface in redox mediator 5 mM FcMeOH (ferrocene methanol) in support electrolyte 0.05 M K_2_SO_4_ with tip voltage *V_t_* = +0.25 V and substrate voltage *V_s_* = −0.4 V. The corresponding theoretical fitting is also plotted as dash curves.

**Figure 8:**
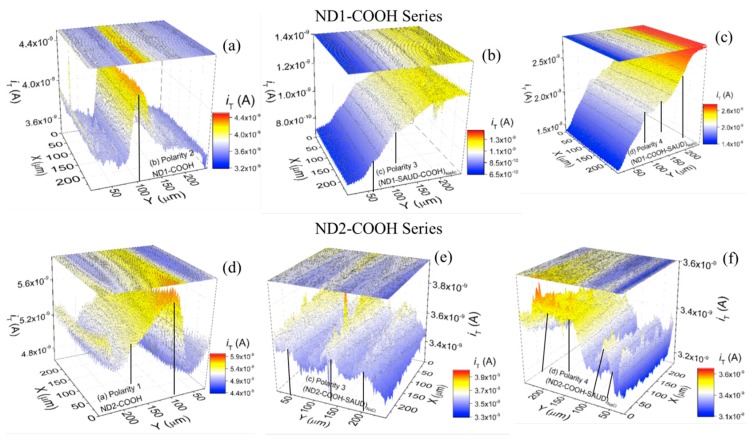
(**a**–**f**) Representative SECM images in 250 × 250 μm^2^ area of unprocessed and processed nanodiamond samples (ND1–COOH and ND2–COOH series) displaying tip current distribution in two- and three-dimensional heat maps with occasional higher (peak)/lower (valley or almost flat) current i.e., highly electroactive regions or ‘hot spots’ marked with vertical lines. A color bar is shown for quantitative values of the tip current.

While CV properties characterize effective electrode areas comparable to geometric areas, for detecting redox reactions occurring in the small region in close proximity to the electrode surface (probe approach mode), SECM is used to obtain local quantitative information about electrochemical reaction rates (see Figure 7), image chemical reactivity, and to determine adsorption site density (Figure 8) [12,61,70,71]. The visualization of electrochemical activity is performed in feedback mode taking advantage of positive feedback (conductive/electrochemically active areas) areas versus negative feedback (insulating/semiconductive/relatively electrochemically inactive areas) from the electrode surface. Figure 7 provides probe approach curves displaying normalized tip ion current (*i*_T_/*i*_T_, ∞) with normalized distance, *L* = d/a, where d is the substrate (electrode)-tip distance and a, the radius of the tip. The tip current (*i_T_*) reaches asymptotic behavior with steady-state current following: $\;i_{T,\infty }=4nFCDa$, where *n* is the number of electrons transferred at the electrode tip (O + ne^−^ → R), and *D* is the diffusion coefficient limited by the hemispherical region around the tip in contrast to the planar region for traditional macro-electrode configuration. With a tip approaching conductive heterogeneous electrode surface, the reduced species formed at the tip is oxidized, yielding an increase in tip current (*i_T_* >*i_T_*, ∞, $i_{t}^{cond}(L)=\frac{i_{T}}{i_{T,\infty }}=[k_{1}+k_{2}/L+k_{3}*\exp(k_{4}/L)$) and creating a regenerative “positive” feedback loop. The opposite effect is observed when the probing insulating (or semiconducting) surface and diffusion to the electrode is inhibited as a result of physical obstruction as the tip approaches the substrate, creating a “negative” feedback loop and decreasing the tip current (*i_T_* <*i_T_*,∞, $i_{T}^{ins}(L)=\frac{i_{T}}{i_{T,\infty }}=1/[k_{1}+k_{2}/L+k_{3}*\exp(k_{4}/L)$). Alternatively, by changing the polarity of the tip with respect to the working electrode (or substrate) we can achieve the reverse scenario. Consequently, the total tip current is given by: $i_{T}=\frac{nFD}{d}C\pi a^{2}+4nFDCa$, where the symbols have the usual meaning. Following measurements, the fitting of the probe approach curves (plotted as dashed curves in Figure 7) for all of the NDs is performed with an accuracy of ~1% which is smaller than typical experimental uncertainties. The heterogeneous rate transfer constant (k_1_, which is the first fitting parameter is for single-electron behavior) values ranged from 5.6 × 10^−2^ cm s^−1^ to 0.1 × 10^−2^ cm s^−1^ for FcMeOH^+^, which are 1000 times smaller than those for the bare Au electrode [70,71,72]. Figure 8 displays SECM area scans in two- and three-dimension heat maps, where the probe (or tip) current is graphed. The tip was polarized at sufficient potential to cause an electrochemical redox reaction (generator) and the current was recorded (collected) over the polarized electrode surfaces. The SECM imaging exhibits pronounced electroactive regions ”hot spots” in SAUD processed NDs and air-oxidized ND–COOH samples. The higher/lower current over the electrodes surface (sites marked with vertical lines in Figure 8) are characteristic of semiconducting behavior at the solid/liquid interface. It is apparent from the current distribution that the ND samples yielded several regions of highly electroactive sites density (full-width at half maximum of ~20–40 μm) reinforcing the multiple roles played by nanoscale size and surface functionality of NDs in contrast to their bulk counterpart.

## 4. Conclusions

In summary, the salt-assisted ultrasonicated de-aggregation process is used for nanodiamond to produce single digit well-dispersed colloid suspensions. A range of analytical techniques revealed a monodispersed state, high phase purity with well-defined crystalline structure, fluorescence emission in the visible region, quantum size effects as well as providing insights into the surface redox chemistry behavior at the nanoscale. Therefore this method proved to be efficient by virtue of electrostatic stabilization and the processed nanodiamond particles become suitable for nanocomposites, electrocatalysis, bio-labeling, and other biomedical applications. The results were compared with unprocessed (air oxidized) and as-received nanodiamond samples. The electron transfer kinetics and diffusion coefficient of ND particles signifies the apparent predominance of surface states as acceptors and surface antibonding character thereby supporting strong electrocatalytic redox processes in the presence of redox-active molecules. Furthermore, the redox electroactivity mapping i.e., the imaging probe ion current distribution, indicated regions of higher electroactive sites distribution ‘hot spots’ at the electrode/electrolyte interface signifying the accessibility of core-shell interfaces (and/or carbon bonding interphases), and edge plane sites of de-aggregated isolated nanodiamond particles as opposed to agglomerated or clustered nanodiamond.

## Figures and Tables

**Table 1:** The estimated flat band potentials (*E*_f_) and carrier concentration (*N*) for all the nanodiamond samples in 0.05 M K_2_SO_4_ solution.

Sample	E_f_ (V vs. Ag/AgCl)	N (cm^−3^)
ND1–COOH	1.02	1.2 × 10^20^
ND2–COOH	1.00	3.5 × 10^21^
(ND1–COOH–SAUD)_NaCl_	0.91	0.5 × 10^21^
(ND1–COOH–SAUD)_NaAc_	0.99	4.07 × 10^21^
(ND2–COOH–SAUD)_NaCl_	0.90	5.1 × 10^21^
(ND2–COOH–SAUD)_NaAc_	0.71	6.2 × 10^21^

## Supplementary Materials

The following are available online at www.mdpi.com/1996-1944/10/11/1292/s1. The supplemental material provides dynamic light scattering experimental data (smoothed using Origin software v. 2017) for unprocessed and SAUD processed NDs estimating the average particle size. Figure S1: Particle size distribution of initial (red) and SAUD processed (green and blue) nanodiamond aqueous dispersions at 2.5 wt % concentrations calculated using UV–Vis absorption: (a) ND1–COOH, ND1–COOH_NaCl_, ND1–COOH_NaAc_ and (b) ND2–COOH, ND2–COOH_NaCl_, ND2–COOH_NaAc_.
