# Supplementary material for: Salt-Assisted Ultrasonicated De-Aggregation and Advanced Redox Electrochemistry of Detonation Nanodiamond
Source: Materials (Basel). 2017 Nov 10;10(11):1292. doi: 10.3390/ma10111292 (PMC5706239; doi:10.3390/ma10111292)
Supplement: Supplementary file 1 [file materials-10-01292-s001.pdf]

Supplementary Materials

Salt-Assisted Ultrasonicated De-Aggregation and Advanced Redox Electrochemistry of Detonation Nanodiamond

Sanju Gupta[1,*], Brendan Evans[1], Alex Henson[1] and Sara B. Carrizosa[2]

[1] Department of Physics and Astronomy and Biotechnology Center, Western Kentucky University, Bowling Green, KY 42101

[2] Department of Chemistry, Western Kentucky University, Bowling Green, KY 42101

Experimental

For ND1-COOH, an average size of 9 ± 3 nm was obtained with 80% of the particles within this range, post SAUD processing with sodium chloride (NaCl) salt and 14 ± 3 nm for sodium acetate (CH_3COONa) salt. Likewise, for ND2–COOH, an average size of 10 ± 3 nm was obtained with 85% of the particles within this range post SAUD processing with sodium chloride (NaCl) salt and 19 ± 3 nm for sodium acetate (CH_3COONa) salt. As suggested by transmission electron microscopy (TEM) images, the nanodiamond nanoparticles tend to adhere and form min-clusters due to weak van der Waals bonding and it is the size of these small clusters that is typically measured by dynamic light scattering (DLS) technique. Due to this coalescence effect, the individual detonation particles are actually of smaller diameter than those provided by DLS. The clustering effect is also observed using the Tyndall effect.

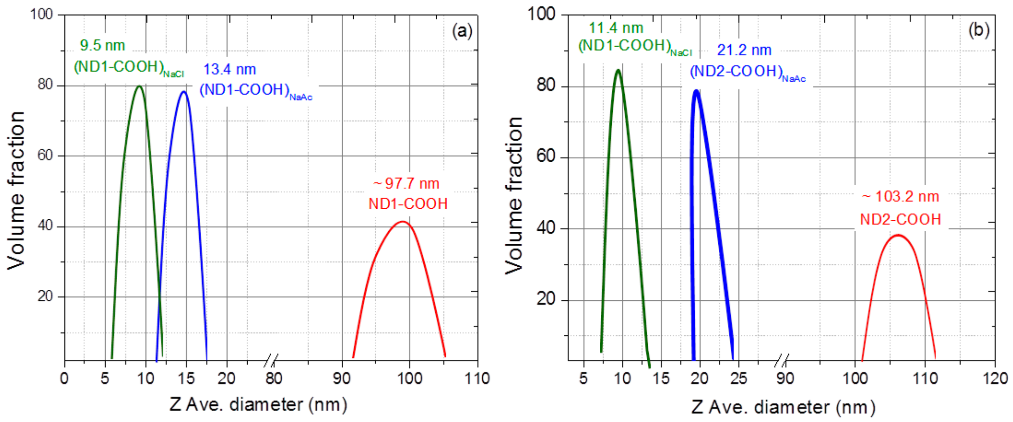

Figure S1. Power spectral density (PSD) showing particle size distribution of initial (red) and SAUD processed (green and blue) nanodiamond aqueous dispersions at 2.5 wt% concentrations calculated using UV–Vis absorption: (**a**) ND1–COOH, ND1–$COOH_{NaCl}$, ND1–$COOH_{NaAc}$ and (**b**) ND2–COOH, ND2–$COOH_{NaCl}$, ND2–$COOH_{NaAc}$.

* The author to whom correspondence should be made. E-Mail: sanju.gupta@wku.edu.

One of the most important physical properties of particulate samples is particle size. The particle size distribution was evaluated by the dynamic light scattering (DLS) technique (Zetasizer Nano Range, Malvern Instruments Inc. Westborough MA), see Figure S1 above. The detection of size population was possible using Taylor Dispersion Analysis (TDA). Briefly, TDA is a micro-capillary flow technique and the working principle is by measuring the time-evolved concentration profile (or Taylorgram) of nanoliter volume sample pulse injected into a laminar flow of matched buffer or dispersant. The sample pulse broadens as it flows along the microcapillary due to dispersion (axial direction) and diffusion (radial direction). UV detection at fixed windows along the micro-capillary is used to analyze the UV absorbance at cross-sections of the sample pulse. Absorbance is plotted as a function of time to produce a Taylorgram and the width is related to the diffusion coefficient (D) of the solute species in the sample and hence the radius or diameter is determined. Taylor Dispersion Analysis with UV area imaging detection offers mass-weighted sizing measurements that are not affected by the presence of a small amount of aggregates, meaning samples can be run without dilution or filtration.
